# Comparative Effectiveness of Complex Telemedicine Support in Prevention of Hospitalizations and Mortality in Patients with Heart Failure: A Systematic Review and Meta-Analysis

**DOI:** 10.3390/life14040507

**Published:** 2024-04-15

**Authors:** Andrey Garanin, Anatoly Rubanenko, Yuriy Trusov, Olesya Rubanenko, Alexandr Kolsanov

**Affiliations:** 1 Scientific and Practical Center for Remote Medicine, FSBEI HE SamSMU MOH Russia, 443099 Samara, Russia; a.a.garanin@samsmu.ru; 2 Propaedeutic Therapy Department with the Course of Cardiology, FSBEI HE SamSMU MOH Russia, 443099 Samara, Russia; yu.a.trusov@samsmu.ru; 3 Hospital Therapy Department with Courses of Transfusiology and Polyclinic Therapy, FSBEI HE SamSMU MOH Russia, 443099 Samara, Russia; o.a.rubanenko@samsmu.ru; 4 Operative Surgery and Clinical Anatomy Department with the Course of Medical Information Technologies, FSBEI HE SamSMU MOH Russia, 443099 Samara, Russia; a.v.kolsanov@samsmu.ru

**Keywords:** heart failure, telemedicine, telemonitoring, hospitalization, mortality

## Abstract

Heart failure is one of the leading causes of hospitalizations and mortality all over the world. There are literature data about the favorable influence of telemedicine support on mortality and hospitalization rate in patients with heart failure, and thus, the results of different studies are controversial. Aim: To estimate the effect of telemedicine support on hospitalization and mortality in patients with heart failure. Methods: The literature search was conducted in databases Google Scholar, MedLine, Clinical Trials, PubMed, Embase, and Crossref with the following key words: “heart failure”, “telemedicine”, “telemonitoring”, “hospitalisation (hospitalization)”, “mortality”. We included studies that were conducted during the last 10 years. In total, we analyzed 1151 records. After screening, 14 randomized control trials were included in the final analysis. Results: The conducted meta-analysis showed that telemedicine support is accompanied by a decrease in heart failure-related hospitalizations (risk ratio (RR) 0.78 (95% confidence interval (CI) 0.68–0.89)) and a decrease in all-cause mortality (RR 0.84 (95% CI 0.75–0.94)). We did not find a significant association between telemedicine support and all-cause hospitalizations. We did not analyze heart failure-related mortality because of insufficient data. Conclusion: Telemedicine support is accompanied by a decrease in heart failure-related hospitalizations and a decrease in all-cause mortality in patients with heart failure.

## 1. Background

The prevalence of heart failure (HF) is currently about 1–2% in adults [1]. Since the abovementioned prevalence shows only diagnosed cases of HF, we expect the true prevalence to be higher [1]. Like in different cardiovascular diseases, the prevalence of HF increases with age [2]. Although the prognosis of patients with HF has improved over the last few decades, especially in patients with heart failure with reduced ejection fraction, it still remains poor and mortality rate still remains pretty high. The 1-year and 5-year mortality rates after making a diagnose of all types of HF were found to be 20% and 53%, respectively, in the Olmsted County Cohort, Minnesota, from 2000 to 2010 [3]. According to data of Shah K.S. et al. (2017) the 5-year mortality of patients with HF was 75.3–75.7% for all types and the median survival was 2.1 years [4]. The high mortality rate of patients with heart failure has been demonstrated in a study that combined Framingham Heart Study and Cardiovascular Health Study cohorts. The authors found that 5-year mortality rate in abovementioned cohorts was 67% [5]. Left ventricle ejection fraction is one of the factors associated with mortality in patients with heart failure. Patients with mildly reduced ejection fraction have a better prognosis compared to patients with a reduced ejection fraction [6]. Of course, there may be a lot of other factors that can have an influence on the prognosis of patients with heart failure. Some of them can worsen the prognosis, and others can improve it. One of the factors that can be beneficial in patients with HF for improving their prognosis is telemedicine support.

Telemedicine is a rapidly growing branch of medicine that has many advantages such as remote patient consultations, remote estimation of vital parameters such as blood pressure, heart rate, body temperature, respiration rate, body mass and others. Telemedicine support may improve patient’s medical education, adherence to treatment, and quality of life; hence, it can be used in complex treatment and preventive strategies in patients with HF. Nowadays there are different telemedicine support systems that can be used in patients with HF. They can include implantable devices (CardioMEMS, ICD/CRT devices) and non-invasive systems (telemonitoring) [7]. A CardioMEMS device is implanted into the pulmonary artery, and then it measures and transmits pulmonary artery pressure data to a local telemedicine center [7]. Telemonitoring can be performed in patients with ICD/CRT devices. These devices can collect information about tachyarrhythmia, the number of ventricular extrasystoles, intracardiac electrogram dynamics, patient activity, etc. [7]. The abovementioned parameters can be checked remotely in manual or automatic mode. A non-invasive approach for patients with HF can include a voice response system which collects information from the patients, electronic scales with automatic transmission of the results to the doctor, and different systems that can collect information about blood pressure, heart rate, respiratory rate, oxygen saturation, virtual visits [8], etc. Mariani et al. demonstrated that virtual visits had the same feasibility and effectiveness compared to in-person visits and delivered higher patient satisfaction [8].

Nowadays, there are many different digital solutions (digital tools, applications, etc.) for telemedicine that usually require special training in order to implement them in clinical practice. Targeted programs will enhance healthcare professionals’ skills and can contribute to further improvement of the management of cardiovascular diseases, particularly HF [9].

To date, many studies have been conducted in order to reveal the influence of telemedicine support on mortality in patients with HF. Some meta-analyses have revealed that telemedicine is associated with a reduction in mortality in patients with HF [10]. At the same time, some other meta-analyses do not demonstrate that telemedicine support can significantly reduce HF-related mortality or all-cause mortality in patients with HF [11,12]. It is important to mention that in many meta-analyses, HF-related mortality was not analyzed as an outcome or it was included in a composite endpoint.

Patients with HF usually undergo frequent hospitalizations: on average, they are hospitalized at least one time per year [13]. In the Olmsted County cohort, the mean rate of hospitalizations in patients with HF was 1.3 per person-year [3]. As far as HF hospitalizations increase with age, and also in patients with comorbidities such as diabetes and kidney diseases, the number of hospitalizations in patients with HF is expected to increase in the future. The impact of telemedicine support on the rate of HF-related hospitalizations is pretty consistent according to modern literature data. Most of the existing meta-analyses reveal a decrease in HF-related hospitalizations in patients with HF and telemedicine support [10,12,14]. But one of the most important problems relating to the abovementioned topic is that most of these meta-analyses show a decrease in HF-related hospitalizations in patients with HF and telemedicine support, while data about mortality remain controversial. In order to clarify the role of telemedicine support in reducing the mortality and hospitalizations in patients with HF, we planned to conduct the present systematic review and meta-analysis.

The aim of the present systemic review was to estimate the effect of telemedicine support on hospitalization and mortality in patients with heart failure.

## 2. Methods

The literature search was conducted in databases Google Scholar, MedLine, Clinical Trials, PubMed, Embase, Crossref with the following key words: “heart failure”, “telemedicine”, “telemonitoring”, “hospitalisation (hospitalization)”, “mortality”. We included studies that were conducted during the last 10 years. The authors independently selected relevant studies. Disagreements between the authors were resolved by consensus. In the final analysis, we included only randomized control trials in which one of the groups of the patients with heart failure received standard treatment and the other group received standard treatment and had telemonitoring support. We included trials with the following endpoints: all-cause mortality, HF-related mortality, all-cause hospitalizations, HF-related hospitalizations, and cardiovascular disease-related hospitalizations. We also included trials with composite endpoints which include mortality or hospitalizations.

Statistical data processing was performed using Review Manager (RevMan), version 5.4.1 (The Cochrane Collaboration, 2020). Meta-analysis was performed according to the model of fixed effects with index of heterogeneity I2 < 40% and random effects with index of heterogeneity I2 > 40% with the inverse variance method. Results of meta-analysis were presented by forest plot. The estimation of statistical heterogeneity was performed with Pearson’s Chi-square, *p* < 0.10. Meta-analysis of absolute indicators in both of the groups was performed with the data of absolute meanings paying attention to the quantity of the patients in compared groups. We estimated the result of telemonitoring with risk ratio with 95% confidence interval (CI). The estimation of risk of bias of individual studies that were included into the systematic review was performed with the Cochrane Collaboration tool for assessing risk of bias. General risk of bias was estimated according to 6 domains: random sequence generation, allocation concealment, blinding of patients and personnel, blinding of outcome assessment, incomplete outcome data, and selective reporting.

## 3. Results

**Figure 1:**
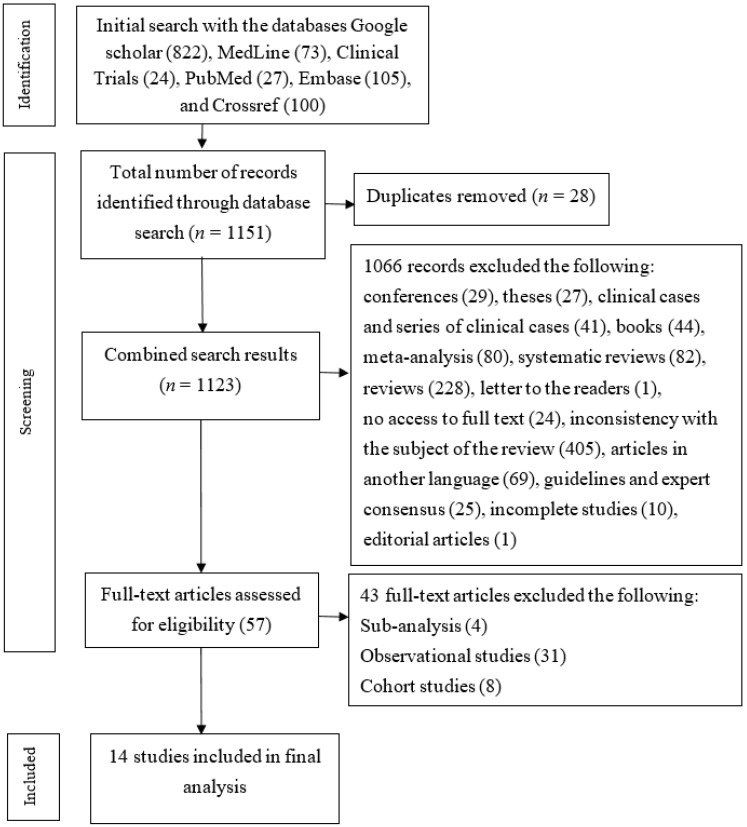
PRISMA flow diagram of studies selection.

During the systematic search, we found a total of 1151 records. After adjustment for duplicates, 1123 records remained. After screening of the title and the abstract, 1066 records were excluded. The main reason for the exclusion of 24 articles was that we did not have full-text access and that they were not randomized control trials according to their abstract. After a full-text review, 14 randomized control trials met the inclusion criteria for the present systematic review. A PRISMA flow diagram of study selection is shown in Figure 1. Main characteristics of included studies are shown in Table 1.

**Figure 2:**
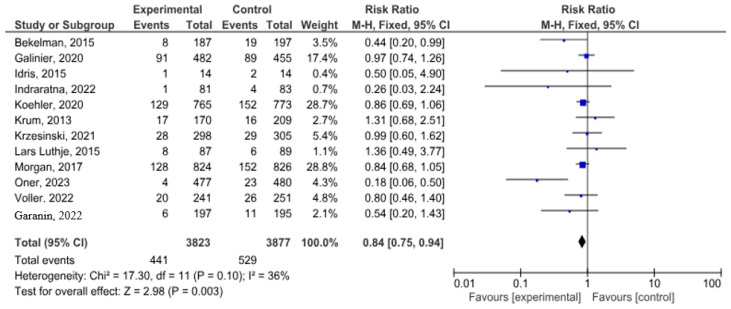
All-cause mortality in patients with HF [15,16,17,19,20,21,22,23,24,26,27,28].

In a meta-analysis devoted to all-cause mortality in patients with HF, 12 studies were included with a total of 7700 patients. Almost half of these patients underwent telemonitoring—3823 (49.6%). All-cause death happened in 441 patients with telemedicine support and in 529 patients without telemedicine support (risk ratio 0.84, 0.75–0.94, *p* = 0.003; I2 = 36%) (Figure 2).

**Figure 3:**
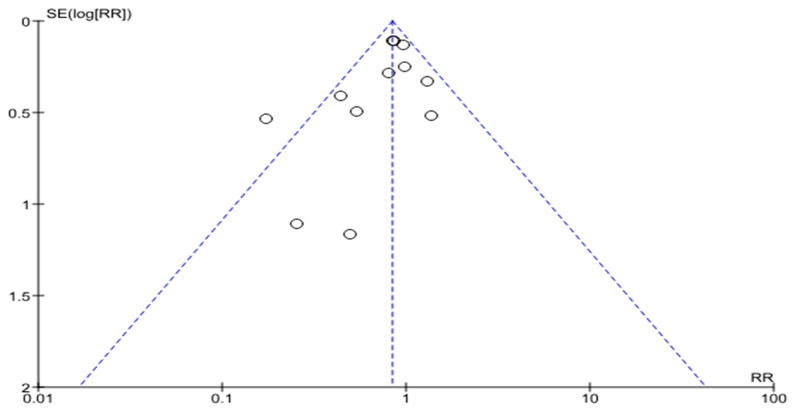
All-cause mortality in patients with HF (funnel plot).

The funnel plot is shown in Figure 3. We can see the variation in the size of the effects for patients in the presented RCTs relative to the axis of the central trend. There is also some asymmetry of the funnel-shaped scattering diagram with a significant number of studies included in the analysis.

**Figure 4:**
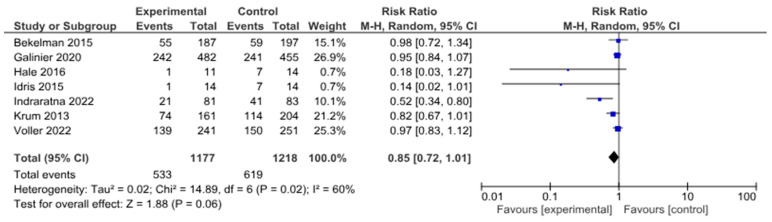
All-cause hospitalizations in patients with HF [15,21,23,25,26,27,28].

In a meta-analysis devoted to all-cause hospitalizations in patients with HF, seven studies were included with a total of 2395 patients. Almost half of them underwent telemonitoring—1177 (49.1%). All-cause hospitalizations happened in 533 patients with telemedicine support and in 619 patients without telemedicine support (risk ratio 0.85, 0.72–1.01, *p* = 0.06; I2 = 60%) (Figure 4).

**Figure 5:**
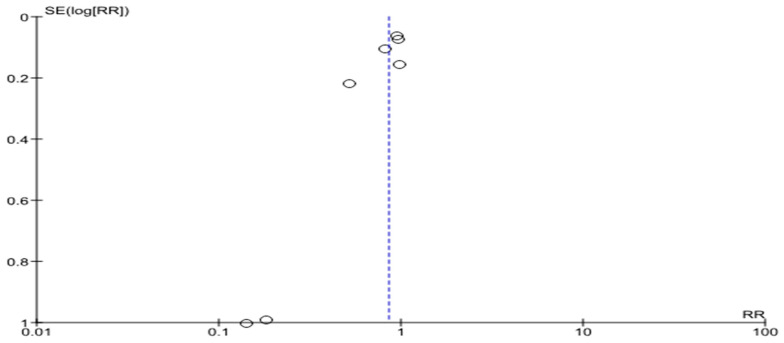
All-cause hospitalizations in patients with HF (funnel plot).

The funnel plot is shown in Figure 5. We can see a significant variation in the size of the effects for patients in the presented RCTs relative to the axis of the central trend. There is a pronounced asymmetry of the diagram with a small number of analyzed studies.

**Figure 6:**
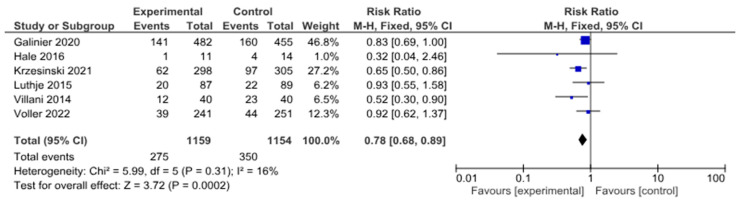
HF-related hospitalizations in patients with HF [16,18,19,21,25,26].

In a meta-analysis devoted to HF-related hospitalizations, six studies were included with a total of 2313 patients. Half of them underwent telemonitoring—1159 (50.1%). HF-related hospitalizations happened in 275 patients with telemedicine support and in 350 patients without telemedicine support (risk ratio 0.78, 0.68–0.89, *p* = 0.0002; I2 = 16%) (Figure 6).

**Figure 7:**
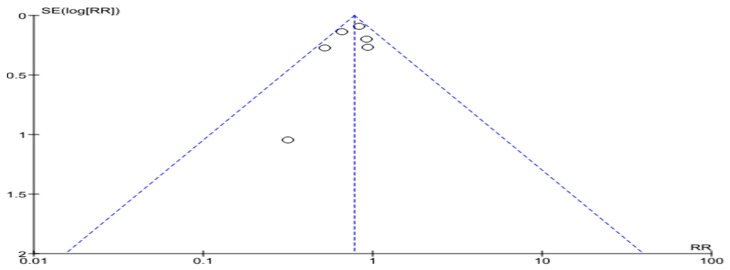
HF-related hospitalizations in patients with HF (funnel plot).

The funnel plot is shown in Figure 7. There was also a variation in the size of the effects for patients in the presented RCTs relative to the axis of the central trend, a significant asymmetry of the diagram with a small number of studies.

**Figure 8:**
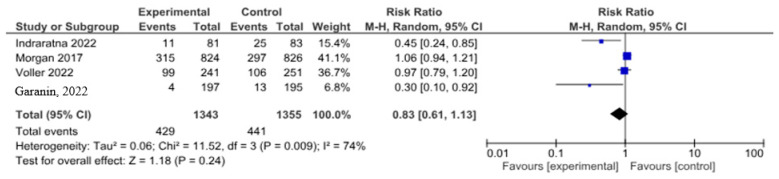
Cardiovascular disease-related hospitalizations in patients with HF [17,20,23,26].

In a meta-analysis devoted to cardiovascular disease-related hospitalizations, four studies were included with a total of 2698 patients. Half of them underwent telemonitoring—1343 (49.8%). Cardiovascular disease-related hospitalizations happened in 429 patients with telemedicine support and in 441 patients without telemedicine support (risk ratio 0.83, 0.61–1.13, *p* = 0.24; I2 = 174%) (Figure 8).

**Figure 9:**
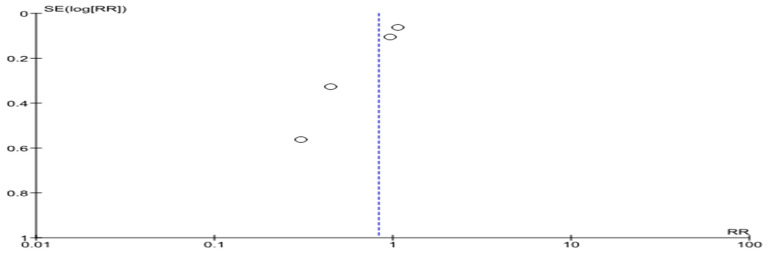
Cardiovascular disease-related hospitalizations in patients with HF (funnel plot).

Figure 9 demonstrates the asymmetry of the funnel-shaped scattering diagram with a small number of included studies.

We could not conduct meta-analysis devoted to HF-related mortality because of insufficient data relating to this endpoint in the abovementioned studies.

## 4. Assessment of the Risks of Bias

**Figure 10:**
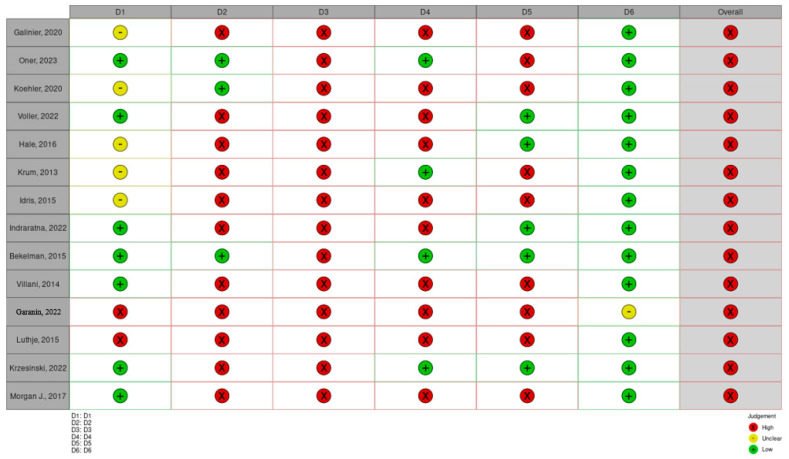
Assessment of the risks of bias of the included RCTs. D1, random sequence generation; D2, allocation concealment; D3, blinding of patients and personnel; D4, blinding of outcome assessment; D5, incomplete outcome data; D6, selective reporting [15,16,17,18,19,20,21,22,23,24,25,26,27,28].

**Figure 11:**
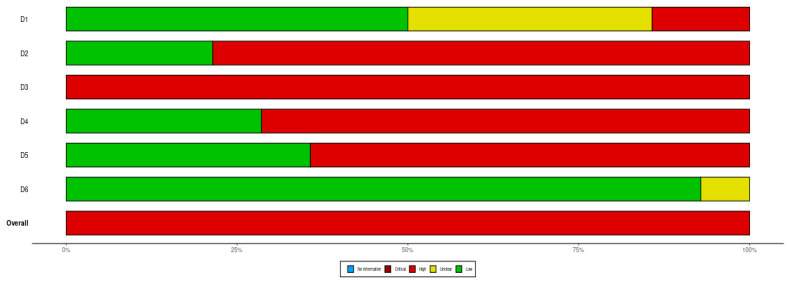
Illustration of the risks of bias of the included randomized control trials. D1, random sequence generation; D2, allocation concealment; D3, blinding of patients and personnel; D4, blinding of outcome assessment; D5, incomplete outcome data; D6, selective reporting.

The assessment of the risk of bias of the included randomized control trials was carried out in accordance with Cochrane Collaboration’s tool for assessing the risk of bias. Overall, 7 studies had a random sequence generation, 3 studies featured allocation concealment, none of the studies featured blinding of patients and personnel, 4 studies utilized blinding of outcome assessment, 5 studies had incomplete outcome data, and 13 studies had selective reporting. In total, all of the studies had a high risk of bias (Figure 10 and Figure 11).

A study conducted by Beckelman et al. from 2009 to 2011 enrolled 392 patients with HF. Patients were represented mainly by males (96.6%), with an average age of 68 years. The subjects were divided into two groups: intervention group and control group. The intervention group had patient-centered disease management that also included home telemonitoring. The intervention group had significantly fewer deaths during year 1 (8 out of 187 [4.3%]) compared to the control group (19 out of 197 [9.6%]) (*p* = 0.04). Patients in the intervention group had less mortality (*p* = 0.04, log-rank test). There was no significant difference in the rates of hospitalization and the length of hospital stay during year 1 (29.4% vs. 29.9%, *p* = 0.87) [15].

The systematic review conducted by Lee K.C. et al. implied an analysis of three categories in “virtual” medicine (telemonitoring, remote patient management, and patient self-monitoring). The authors concluded that an integrated approach to remote patient management solutions is the most effective solution in reducing the frequency of repeated hospitalizations of patients with HF and the total number of hospital visits. A positive result was recorded in predicting the outcomes of HF [29].

Garanin et al. in a study of 392 patients with chronic heart failure (CHF), randomized them into active observation group by remote blood pressure monitoring (group 1, *n* = 197) and standard management group (group 2, *n* = 195), conducted over 3 months. A total of 4 patients from group 1 required hospitalization, which was associated with decompensation of CHF or an episode of acute coronary syndrome with a total duration of 30 days, compared with 13 hospitalizations for the same reasons in group 2 (*p* = 0.027; OR = 3.4; 95% CI 1.1–10.8) [17].

A randomized multicenter OSICAT study enrolled 937 patients hospitalized for acute HF. Patients were randomized to a telemonitoring group (daily body weight measurement, daily registration of HF symptoms and individual training) (*n* = 482) or to a control group (*n* = 455). As a result of this study, the authors did not record a significant decrease in mortality or a decrease in the risk of repeated hospitalizations. It is worth noting that in the telemonitoring group, there was 21% decrease in the risk of first unplanned hospitalization for HF (OR 0.79, 95% CI 0.62–0.99; *p* = 0.044). The relative risk reduction was 29% in patients with CHF III or IV NYHA class (OR 0.71, 95% CI 0.53–0.95; *p* = 0.02), 38% in socially isolated patients (OR 0.62, 95% CI 0.39–0.98; *p* = 0.043), and 37% in patients who underwent constant body weight measurements (OR 0.63, 95% CI 0.45–0.88; *p* = 0.006) [21].

However, the results obtained by Hale et al., who investigated the use of a remote monitoring system for taking medications for 90 days on 25 subjects randomized to a control group (14 people) and an intervention group (11 people), demonstrate an 80% reduction in the risk of hospitalization and a reduction in the length of hospital stay in the intervention group. The assessment of the quality of life revealed the opposite—the participants in the intervention group had a lower level of quality of life compared to the control group, which was probably associated with an initially more severe HF in the first group [25].

Idris et al. conducted an integrated telemonitoring study, which included 24 patients with NYHA class II/III and left ventricular ejection fraction (LVEF) < 35%. Patients were randomized in a 1:1 ratio to the control or intervention group. The control group received a standard treatment of beta-blockers, angiotensin-converting enzyme inhibitors, aldosterone receptor antagonists, vasodilators, and diuretics, dosed to achieve resting heart rate < 100 beats per minute and blood pressure < 140/90 mmHg. The intervention group received the standard treatment, as well as additional daily monitoring of blood pressure, heart rate, blood saturation oxygen, and weight using the Health Connect telemonitoring system for 3 months. In addition, weekly specialist consultation calls were organized in the intervention group. There was no significant difference in concomitant diseases, NT-proBNP level (7094 vs. 9994, *p* = 0.33), and ejection fraction (23.5 vs. 22.1 *p* = 0.4) between participants in both study groups. In the first 3 months of the study, the patients in the intervention group received weekly telemedicine consultations in the form of teleconferences. The rates of recurrent hospitalizations in the first 30 days had a statistically significant difference (1 vs. 7, *p* = 0.03). However, the analysis of the same indicator after 3 and 6 months did not reveal significant differences between the two groups. The mortality rate during the 12 months of the study was 7% in the intervention group and 14% in the control group (*p* > 0.99) [28].

An Australian randomized control trial included 164 patients (130 of whom were male), divided into a group using telemedicine technologies 81/164 (49.4%) and a control group 83/164 (50.6%). The average age of the subjects was 61.5 years. During the study, 11 unplanned hospitalizations lasting 30 days were recorded in both groups (*p* = 0.97) [23]. During 193 days of follow-up, a significant reduction in unplanned repeated hospitalizations was revealed (21 in the intervention group versus 41 in the control group; *p* = 0.02), including cardiovascular disease-related recurrent hospitalizations (11 in the first versus 25 in the second group; *p* = 0.03), as well as improvements in the completion rate of cardiac rehabilitation (20/51 (39%) vs. 9/49 (18%), *p* = 0.03), which is consistent with the data of Rosario et al. [30].

To the best of our knowledge, only one randomized control trial using mHealth has demonstrated a decrease in the number of repeat hospitalizations in patients with HF. In a study by Dendale et al., 160 patients with HF took part, and a similar method of data transmission was utilized. On average, over a 6-month period, patients submitted 27 warnings about the deterioration of their current condition, which is higher than in the study by Indraratna et al. [23,31].

The multicenter TIM-HF2 study, which took place in 43 hospitals and 60 cardiological clinics in Germany, included patients with HF II and III NYHA class, hospitalized over a 12-month period. The patients were randomized into a remote patient management (RPM) program participation group and a control group. At the final visit, the RPM intervention was stopped, after which a period of extended follow-up began, which lasted 1 year. The study included 1538 patients (765 in the remote patient management group and 773 in the control group). Overall, 671 out of 765 patients from the first group and 673 out of 773 patients from the second group completed the main study and began an extended follow-up period 1 year later. The percentage of days spent on unplanned hospitalization for CVD and mortality from all causes in the extended follow-up period did not differ significantly between the groups: 5.95% [95% CI 4.59–7.31] in the remote management group versus 6.64% [95% CI 5.19–8.08] in the conventional treatment group [OR 0.79; 95% CI 0.78–1.21]). However, when combining the data from the main study and the extended follow-up period, the percentage of days of unplanned cardiovascular hospitalization or all-cause death was significantly lower in patients included in the remote patient management group (382 [50%] of 765; weighted mean 9.28%; 95% CI 7.76–10.81) than in the control group (398 [51%] of 773; 11.78%; 95% CI 10.08–13.49; ratio of weighted average 0.79; 95% CI 0.62–1.00; *p* = 0.0486) [22].

Krum et al. conducted a study of patients with HF, randomized into groups with conventional treatment (control group) and intervention group with telephone support (TS) (intervention group), with the participation of 143 general practitioners across Australia. The follow-up period of the patients was 12 months. The subjects underwent a Packer clinical composite score, which was the primary endpoint. Secondary endpoints included hospitalization for any reason, death, or hospitalization for HF. A total of 405 patients were randomized into the TS group. The results of the Packer scale assessment showed no differences between the two groups (*p* = 0.98), although in the TS group, there was some improvement in a larger number of patients. In the intervention group, compared with the control group, there were fewer patients hospitalized for any cause (74 vs. 114, OR 0.67 [95% CI 0.50–0.89], *p* = 0.006) and also who died or were hospitalized (89 vs. 124, OR 0.70 [95% CI 0.53–0.92], *p* = 0.011). The number of HF-related hospitalizations decreased when using TS (23 vs. 35, OR 0.81 [95% CI 0.44–1.38]), (*p* = 0.43). There were 16 deaths in the control group and 17 in the TS group (*p* = 0.43). At the same time, patients in the control group visited the physician more often than patients from TS (12.55 vs. 5.85). The values of NT-proBNP (median and IQR) at the beginning of the study were 1053 (370–2341) and 1105.5 (367.75–2572.5) for the control groups (*n* = 203) and intervention (*n* = 176). After 12 months, the level of NT-proBNP was 960 (374–2007) and 1083 (408.5–2182.5) for the first (*n* = 123) and second groups (*n* = 117). This study demonstrates that intervention in the form of TS can be an effective approach to improve clinical outcomes in patients with HF in rural and remote areas [27].

In the AMULET study, patients with HF and LVEF ≤ 49% with an episode of acute HF in the last 6 months were randomized to receive outpatient telecare based on non-invasive assessments under the guidance of a nurse (*n* = 300) (AMULET model) or a standard treatment group (*n* = 305). Unplanned hospitalization for HF or death due to cardiovascular pathology was detected in 51 (17.1%) patients in the telecare group and in 73 (23.9%) patients in the control group during the 12 months of the study (OR 0.69, 95% CI 0.48–0.99; *p* = 0.044). The introduction of AMULET telecare, compared with standard treatment, reduced the risk of first unplanned hospitalization for HF (OR 0.62, 95% CI 0.42–0.91; *p* = 0.015). There was no difference in cardiovascular mortality between the study groups (OR 1.03, 95% CI 0.54–1.67; *p* = 0.930) [16]. Researchers report a 31% reduction in the risk of unplanned hospitalization for HF or cardiovascular death in patients after an episode of acute HF. It is also noted that thanks to telemedicine intervention, the total number of all unplanned hospitalizations for HF decreased by 36%.

Lüthje et al. conducted a prospective single-center randomized trial during the period 2007–2011 and enrolled 176 patients. The authors evaluated the effectiveness of remote monitoring in patients with HF [19]. The subjects were randomized to a remote monitoring group including OptiVol and to a control group (subjects were examined every 3 months in the control group). A total of 176 patients were analyzed (77% men; 66 ± 12 years old; LVEF 32 ± 11%; cardiomyopathy 50%; digital implantable cardioverter defibrillator (ICD) 50%; primary prevention 85%). An analysis of the proportional risk model (Cox regression) of the time before the first hospitalization for HF showed that the OR was 1.23 [0.62–2.44] (*p* = 0.551) in favor of the control group. In the remote monitoring group, 13 patients (15%) suffered ICD shock compared to 10 patients (11%) in the control group (*p* = 0.512). The average time to the first ICD shock was 212 ± 173 days in the remote monitoring group and 212 ± 143 days in the control group (*p* = 0.994). The Kaplan–Meier life expectancy estimate after 1 year was 8.6% (8 deaths) in the remote monitoring group versus 4.6% in the control group (6 deaths; *p* = 0.502) [19].

A study by Villani et al. was devoted to telemonitoring and telecare for patients with chronic HF leaving the hospital. The authors randomized 80 patients before discharge from the hospital into two groups: usual care group (40 patients, follow-up at the outpatient department) and integrated management group (40 patients, who used a handheld PDA with follow-up conducted with the monitoring center). The authors concluded that at one-year follow-up, mortality and hospital re-admissions for congestive heart failure were reduced in integrated management patients (*p* < 0.05) [18].

Morgan et al. randomly assigned 1650 patients with HF and cardiac implantable electronic devices for two groups: group of active remote monitoring and group of usual care. Patients were followed for a median of 2.8 years. The incidence of the primary endpoint (death from any cause or unplanned hospitalization for cardiovascular reasons) did not differ significantly between two groups, which occurred in 42.4 and 40.8% of patients, respectively (hazard ratio 1.01; 95% confidence interval (CI) 0.87–1.18; *p* = 0.87). The two studied groups also were not significantly different with respect to any of secondary endpoints (death from any cause, death from cardiovascular reasons, death from cardiovascular reasons and unplanned cardiovascular hospitalization, unplanned cardiovascular hospitalization, and unplanned hospitalization) [20].

A randomized controlled trial by Oner et al. included 960 patients with either atrial fibrillation, heart failure, or treatment-resistant hypertension that were randomized into two groups: group of novel integrated care concept with combination of telemonitoring with the call-center support in addition to guidelines-based therapy (478 patients), and a standard care group (482 patients). The follow-up period was 12 months. The primary endpoint (composite of all-cause mortality, stroke, and myocardial infarction) occurred in 1.5% of patients with telemonitoring support and in 5.2% of patients with standard care (OR: 3.3 [95% CI 1.4–8.3], *p* = 0.009) [24].

Voller et al. performed multicenter randomized control trial and enrolled 621 patients with HF and reduced ejection fraction. Patients were randomized to remote telemonitoring group (*n* = 302) and standard care group (*n* = 319) with a follow-up period of 12 months. The authors observed 20 events in the remote telemonitoring group and 26 events in the standard care group, with no statistical difference between the groups: HR 0.81 (95% CI 0.45–1.45; log-rank *p* = 0.478). The authors came to the conclusion that remote telemonitoring did not impact mortality risk in patients with HF. The authors also did not find a significant difference for all-cause hospitalizations and HF-related hospitalizations between patients of the two groups [26].

## 5. Discussion

Telemedicine technology is very rapidly evolving. Many new digital tools and applications appear every year, leading to greater convenience for patients and doctors as well. Telemedicine and related technologies are progressively implemented into clinical practice, and it is now pretty hard to imagine modern medicine without remote consultations and telemedicine support.

We conducted meta-analysis, which was devoted to analyzing the influence of telemedicine support on mortality and hospitalizations in patients with HF.

A previously performed meta-analysis by Lin et al. [10] supports the view that telemonitoring can improve the outcomes of patients with HF. The authors declared that the association of telemonitoring with patient outcomes depend on the way telemonitoring is conducted. Home-based teletransmission monitoring was accompanied by lower rates of HF-related hospital admission, all-cause mortality, and HF-related mortality. At the same time, nurse-based telephone support care provided much less benefit, resulting in only a reduction in HF-related hospital admission. Studies that were analyzed in our meta-analysis provided different approaches to telemedicine support in patients with HF: home-based telemonitoring, telephone support, integrated approach, etc. Unfortunately, we were unable to analyze the influence of different methods of telemedicine support on hospitalizations and mortality in patients with HF. The abovementioned fact may be one of the reasons for the controversial results of different studies since they use different methods of telemedicine support. Compared to a meta-analysis by Lin et al., we failed to analyze the association of telemedicine support with HF-related mortality due to insufficient data. For our meta-analysis, we included only recent studies that were conducted within a specific period of 10 years (from 2013 to 2023), which may be the reason why we did not find sufficient data for the analysis of HF-related mortality.

A meta-analysis by Zhu et al. [11] also supported telemedicine in patients with HF. The authors conclude that telemedicine intervention reduces the all-cause hospitalization, cardiac hospitalization, all-cause mortality, cardiac mortality, and length of stay in HF patients. Our meta-analysis was less comprehensive; we did not analyze the length of stay and cardiac mortality in HF patients. At the same time, we analyzed HF-related hospitalization instead of cardiac hospitalization. We did not demonstrate a significant favorable effect of telemedicine support on cardiac hospitalization and all-cause hospitalization in patients with HF, which can be explained by the different studies included in our meta-analysis since we included only recent studies.

Another meta-analysis by Rebolledo Del Toro et al. [12] included only telemonitoring strategies using mobile applications. The authors declared that this telemedicine approach reduces HF-related hospitalizations but not all-cause and cardiovascular mortality and not all-cause hospitalizations. Our results differ from their meta-analysis since we found a significant association of telemedicine support not only with a decrease in heart failure-related hospitalizations but also with decreasing of all-cause mortality. One of the reasons for this difference may be that we did not separately analyze different methods of telemedicine support.

The evidence that telemedicine support helps in reducing the number of hospitalizations in patient with HF remains pretty consistent among different studies. As for mortality, this topic remains to be further discussed, because existing data in the literature are quite controversial. The reason for this issue may be that different studies use different approaches to telemedicine support, which can have an influence on the results. Another reason may be that most of the studies included in meta-analyses have a high risk of bias.

Generally, our results support the evidence that telemedicine is an important instrument in the management of patients with HF since it can facilitate a decrease in hospitalizations and mortality. We hope that the results of our meta-analysis could take one additional step in favoring telemedicine support in decreasing mortality of patients with HF.

## 6. Limitations

Our meta-analysis has some limitations. Some studies included in this meta-analysis had composite endpoints, and some studies enrolled a small number of patients. We did not analyze the type of telemonitoring and the type of HF. The abovementioned limitations could have some influence on the results of our meta-analysis.

## 7. Conclusions

Telemedicine support is accompanied by a decrease in heart failure-related hospitalizations and a decrease in all-cause mortality in patients with heart failure.

## Figures and Tables

**Table 1:** Results of systematic review.

Study	Number of Patients (*n*)	Study Groups (*n*)	Study Endpoints	Observational Period	Treatment Group, Mortality	Control Group, Mortality	Treatment Group, Hospitalizations	Control Group, Hospitalizations
Bekelman D.B., 2015 [15]	392	Home telemonitoring (*n* = 187) Traditional monitoring (*n* = 197)	All-cause mortality, all-cause hospitalizations	12 months	8/187	19/197	55/187	59/197
Krzesinski P., 2022 [16]	605	Telemonitoring (*n* = 300) Traditional monitoring (*n* = 305)	All-cause mortality, HF-related hospitalizations	12 months	28/298	29/305	62/298	97/305
Garanin A.A., 2022 [17]	392	Telemonitoring (*n* = 197) Traditional monitoring (*n* = 195)	All-cause mortality, cardiovascular-related hospitalizations	3 months	6/197	11/195	4/197	13/195
Villani A., 2014 [18]	80	Telemonitoring (*n* = 40) Traditional monitoring (*n* = 40)	Mortality, HF-related hospitalizations	12 months	5/40	9/40	12/40	23/40
Lars Luthje, 2015 [19]	176	Remote monitoring (*n* = 87) Traditional monitoring (*n* = 89)	All-cause mortality, HF-related hospitalizations	15 months	8/87	6/89	20/87	22/89
Morgan J.M. [20]	1650	Remote monitoring (*n* = 824) Traditional monitoring (*n* = 826)	All-cause mortality, cardiovascular-related hospitalizations	At mean 2.8 years	128/824	152/826	315/824	297/826
Galinier M., 2020 [21]	937	Telemonitoring (*n* = 482) Traditional monitoring (*n* = 455)	All-cause mortality, HF-related hospitalizations, all-cause hospitalizations	18 months	91/482	89/455	141/482 242/482	160/455 241/455
Koehler F., 2020 [22]	1538	Telemonitoring (*n* = 765) Traditional monitoring (*n* = 773)	All-cause mortality	24 months	129/765	152/773	-	-
Indraratna P., 2022 [23]	164	Telemonitoring (*n* = 81) Traditional monitoring (*n* = 83)	All-cause mortality, all-cause hospitalizations	193 days	1/81	4/83	21/81	41/83
			cardiovascular-related hospitalizations		-	-	11/81	25/83
Oner A., 2023 [24]	960	Telemonitoring (*n* = 478) Traditional monitoring (*n* = 482)	All-cause mortality	12 months	4/477	23/480	-	-
Hale T.M., 2016 [25]	29	Telemonitoring (*n* = 13) Traditional monitoring (*n* = 16)	All-cause hospitalizations,	3 months	-	-	1/11	7/14
			HF-related hospitalizations		-	-	1/11	4/14
Voller H., 2022 [26]	621	Telemonitoring (*n* = 302) Traditional monitoring (*n* = 319)	All-cause mortality, All-cause hospitalizations,	12 months	20/241	26/251	139/241	150/251
			HF-related hospitalizations,		-	-	39/241	44/251
			cardiovascular-related hospitalizations		-	-	99/241	106/251
Krum H., 2013 [27]	313	Telemonitoring (*n* = 188) Traditional monitoring (*n* = 217)	All-cause mortality, all-cause hospitalizations	12 months	17/170	16/209	74/161	114/204
Idris, 2015 [28]	28	Telemonitoring (*n* = 14) Traditional monitoring (*n* = 14)	All-cause mortality, all-cause hospitalizations	6 months	1/14	2/14	1/14	7/14

## Data Availability

Not applicable.
